# Defining “Normal” basal serum tryptase levels: a context-dependent approach to improve diagnostics in systemic mastocytosis

**DOI:** 10.3389/falgy.2025.1592001

**Published:** 2025-05-12

**Authors:** Francesca Crupi, Jessica Caroprese, Francesco Mannelli

**Affiliations:** ^1^ Dipartimento di Medicina Sperimentale e Clinica, Università di Firenze, Firenze, Italy; ^2^ SOD Ematologia, Università di Firenze, AOU Careggi, Firenze, Italy; ^3^ Centro Ricerca e Innovazione Malattie Mieloproliferative (CRIMM), AOU Careggi, Firenze, Italy

**Keywords:** tryptase, mastocytosis, mast cell disorder, KIT, tryptasemia

## Abstract

Serum tryptase level has long been used as a biomarker in clinical practice to suspect mast-cell associated disorders. Basal serum tryptase (BST) above 20 ng/ml represents a minor criterion according to WHO and ICC for the diagnosis of systemic mastocytosis (SM) although normal BST value does not exclude the diagnosis. Nevertheless, BST can be elevated also due to non-SM related diseases as well as hereditary alpha-tryptasemia (H*α*T), an autosomal dominant germline condition that consists in the increase of the number of copies of the *TPSAB1* gene encoding the alpha isoform of tryptase. The prevalence of H*α*T is estimated at around 5% of the general population. Individuals with H*α*T genotype can be asymptomatic; however, some of them can experience a range of symptoms with a large variability in type and severity, posing a problem of differential diagnosis with SM. The increasing awareness on a potentially SM underlying diverse clinical manifestations has led to excessive BST testing by several specialists, a trend that risks over interpreting some borderline results. The interpretation of elevated BST should thus be carefully appraised in specific clinical contexts on individual basis. This review is intended to examine the existing literature on this topic and offers a guide for interpreting the BST to rationalize the application of invasive diagnostic procedures.

## Introduction

Systemic mastocytosis (SM) is a rare haematological disease characterized by the abnormal accumulation and expansion of neoplastic mast cells (MCs) in one or more extracutaneous organs, leading to widely heterogeneous clinical manifestations. The clinical picture is primarily due to the inappropriate release of MC mediators. On the other hand, in advanced variants (AdvSM), massive MCs infiltration and organ dysfunction are observed (1–3).

Due to the frequently blurred presentation of mediator-related symptoms, with large overlap with several non-SM disorders, patients often consult multiple specialists before being diagnosed. A further challenge is represented by the need of invasive diagnostics, inclusive of bone marrow biopsy, to get or reliably exclude the presence of a MC disorder. The reticence of patients, especially when a- or pauci-symptomatic, to undergo such diagnostic path leads clinicians to request tests in PB, i.e., basal tryptase level (BST) and *KIT* variant, the results of which can often be misleading if not properly considered in the specific setting.

On the other hand, the increasing awareness on a potentially SM underlying diverse clinical manifestations has led to excessive BST testing by several specialists, a trend that risks overinterpreting some borderline results. That is even more relevant considering the growing knowledge of hereditary alpha-tryptasemia (HαT), a germline condition causing increase in BST with not fully elucidated clinical consequences (4–6).

The aim of this work is to provide key indications that should raise suspicion of SM and, specifically, to assess the interpretation of BST with the intent of rationalizing invasive diagnostics. We discuss specific contexts in which to integrate the finding of altered BST, highlighting when it may serve as a red flag for diagnosis, and conversely examining situations in which its value alone should not support diagnostic suspicion.

## Updates in diagnosis and subclassification of systemic mastocytosis

Classification and diagnostic criteria of SM were recently refined by the 2022 International Consensus Classification (ICC) (7) and the 5th edition of the World Health Organization (WHO) classification (8) by incorporating the most recent clinical updates and advances especially in molecular genetics (Table 1).

**Table 1:** Updates in classification of mastocytosis according to 2022 ICC and WHO 5th edition.

Variant	WHO 5th Edition	ICC
Cutaneous Mastocytosis (CM)	Maculopapular CM/Urticaria Pigmentosa	Maculopapular CM
	Diffuse CM	Diffuse CM
	Cutaneous Mastocytoma – Isolated – Multilocalized	Mastocytoma of the skin
**Systemic Mastocytosis (SM)** *	Indolent SM	Indolent SM (Included Bone Marrow Mastocytosis)
	Bone Marrow Mastocytosis	
	Smoldering SM	Smoldering SM
	Aggressive SM	Aggressive SM
	SM with an associated hematologic neoplasm	SM with an associated hematologic neoplasm
	Mast cell leukemia	Mast cell leukemia
Mast Cell Sarcoma (MCS)	MCS	MCS

* **Well-differentiated SM (WDSM)** is a variant of systemic mastocytosis not recognized as an independent subtype according to 2022 ICC and WHO 5th Edition.

SM was categorized into five distinct subtypes: indolent (ISM), smoldering (SSM), aggressive (ASM), associated to another haematological/myeloid neoplasm (SM-AHN), and mast cell leukemia (MCL)*.* Although formally not recognized as an independent subtype, the acknowledgement of peculiar features for well-differentiated SM (WDSM) (9), previously described by the Spanish group (10), led to more comprehensive diagnostic criteria to include these forms, often featured by non-canonical *KIT* variants. Meanwhile, both classifications have defined bone marrow mastocytosis (BMM), a separate variant according to the WHO (8) and a subtype of ISM according to the ICC (7).

Demonstration of multifocal infiltrates of tryptase- or CD117-positive MCs (≥15 mast cells in aggregates) in BM or other extracutaneous organs remained the major diagnostic criterion. The combination with which major and minor criteria meet a full SM diagnosis differs slightly between ICC and WHO. While the WHO (8) requires one major and one minor criterion, or three minor criteria, the ICC (7) is less stringent as it requires only the major criterion or three minor criteria.

Furthermore, minor criteria have been revised in both classifications. The aberrant MC phenotype now includes also CD30 expression beside the positivity of CD2 and/or CD25. The presence of any *KIT*-activating variants, in addition to the canonical D816V, has also been considered. As regards BST, in the WHO classification, its value should be adjusted in case of HαT, if known, while for the ICC the value of BST is waived in the context of SM-AHN. B and C findings reflect the burden of the disease and organ impairment, respectively, and continue to be the key for establishing an accurate subclassification of SM clinical variants. While C-findings remained essentially unchanged, B-findings have been slightly modified, as WHO has embedded a variant allele frequency (VAF) >10% of the *KIT* D816V variant (8), and ICC has simplified the definition of cytopenia (not meeting the criteria for C-findings) or cytosis (7).

## Hereditary alpha tryptasemia and mastocytosis: a clinical interaction?

**Figure 1:**
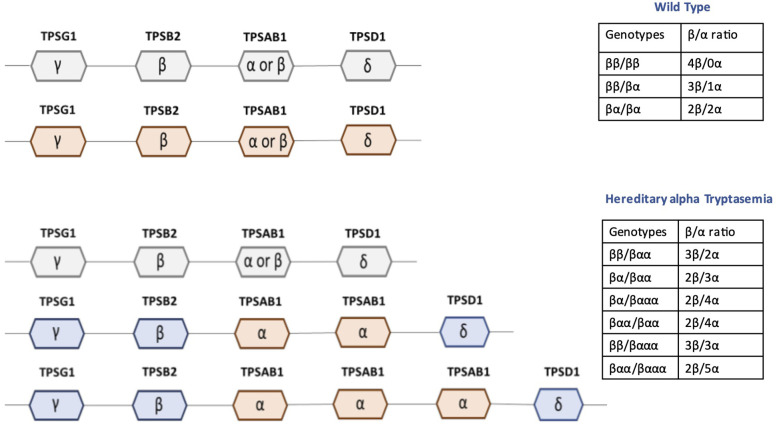
Possible combinations of genotypes for *TPSB2* and *TPSAB1*. Adapted from Sordi et al. JACI, 2023.

Tryptase production depends on the regulation of four genes, among which *TPSAB1* and *TPSB2* encode the two major secreted isoforms, that are α- and β-tryptase. Around a decade ago, Lyons et al. (4, 11) first described a genetic trait known as HαT, an autosomal dominant condition resulting from the multiplication of the *TPSAB1* gene (Figure 1) encoding the alpha isoform of tryptase. Further studies have elucidated as the number of *TPSAB1* copies determined an increase in α-tryptase levels, on turn ultimately leading to elevated BST, each additional copy number adding approximately 9–10 ng/ml to the basal BST levels (12, 13). The increased production of α-tryptase leads to enhanced formation of α/β-tryptase heterotetramers. Recent studies have shown that in individuals with HαT, these heterotetramers increase vascular permeability by cleaving and activating protease-activated receptor-2 (PAR2) on endothelial cells, resulting in increased vascular leakage (14, 15). Furthermore, heterotetramers have been shown to cleave a subunit of the epidermal growth factor-like module-containing mucin-like hormone receptor-like 2 (EMR2) *in vivo* thus causing a decrease in the threshold for vibration-induced MC degranulation (16). These factors have been hypothesized to contribute to hypotension and other systemic reactions in subjects with HαT, potentially accounting for a higher prevalence of HαT among individuals with grade IV Muller anaphylaxis compared to those with less severe forms of anaphylaxis (12, 17). Such correlations are still a matter of debate and need confirmation in larger datasets.

Of interest, the presence of HαT has been investigated as a potential additive trigger for clinical manifestations in SM patients, as severe episodes were twice more frequent in individuals with both SM and HαT than in those with SM but without HαT (17, 18). In spite of a lower burden of disease, HαT+ SM patients displayed a propensity to higher rate of mediator-related symptoms, that supported the role of HαT as a clinical amplifier in this clinical context (19).

These aspects must be considered given the significant prevalence of HαT in the general population, estimated at around 5% (20, 21) and in patients with SM, where it can reach up to 12%–20% (17, 19, 22, 23).

## Tryptase in suspected SM: the interference by HαT

As anticipated, BST is used as one of the main biomarkers in the suspicion of SM and represents a minor criterion for diagnosis when its value exceeds 20 ng/ml (7, 8).

BST normal values have been established in the range of 1 to 11.5 ng/ml (20, 24), maintaining a consistent level in healthy individuals due to the balance between storage and release from MCs (25, 26). However, several reasons other than SM can increase BST, including pregnancy, chronic kidney disease, other myeloid neoplasms and HαT (27).

The need for BST testing and its interpretation thus requires a critical and context-dependent approach for making it a reliable diagnostic tool, while over testing and out-of-context mere consideration of abnormal values risk to be misleading.

In the work-up of anaphylaxis, BST levels raise significantly after the episode due to the considerable release by MC and is expected to turn to the baseline value at least after 24 h (28, 29). The 2012 proposal for mast cell activation syndrome (MCAS) by Valent et al. (30) recommends that an increase in BST levels of more than “20% + 2” (measured within 4 h from symptom onset) should be considered highly suggestive of MCAS. This so-called “20% + 2 formula” was subsequently validated by several studies (31–33). However, a recent study suggested this approach to be not sufficiently performant when applied to patients with HαT and/or SM. These authors proposed an acute tryptase value/BST ratio of 1.685 to improve specificity while maintaining high sensitivity (34). Based on these premises, the need for repeating the tryptase measurement at a reasonable distance from symptom onset, at least two or more days after the event, has clearly emerged (35). If BST value remains persistently elevated and other possible causes are excluded, SM should be suspected, especially if a REMA score of ≥2 is present (36).

The need for operational thresholds for BST (i.e., >20 ng/ml and >200 ng/ml as a minor diagnostic criterion for SM and a B-finding for SSM, respectively) is challenged by the potential presence of an underlying HαT, as it has been well established that the copy number of the *TPSAB1* gene correlates with BST values. A recent proposal (10), also received by the 5th WHO classification (8), suggested normalizing BST value by dividing it by the number of extra copies of the *TPSAB1* gene, encoding alpha tryptase, plus one. In addition to this, three other methods of correction have been proposed (5, 13, 23) (Table 2). All these attempts to adjust the value of BST for HαT are empirical and still to be validated prospectively. In the meantime, to draw a clear line in this debate, the European Competence Network on Mastocytosis (ECNM) and the American Initiative in Mast Cell Diseases (AIM) established that the normal BST range should be set at 1–15 ng/ml, including asymptomatic individuals with H*α*T (35). This proposal thus recommends that, in asymptomatic patients, BST values up to 15 ng/ml should be considered normal to avoid unnecessary referrals and investigations, as well as unjustified worries in interested individuals.

**Table 2:** Available formulae for normalize basal serum tryptase (BST) value according to HαT genotype.

Authors	Year	Journal	Formula
Valent et al.	2021	HemaSphere	BST/(1 + number of extra TPSAB1 copies)
Lyons et al.	2021	Annals of Allergy, Asthma & Immunology	BST−(9 ng/ml) × number of extra TPSAB1 Copies
Chollet et al.	2022	Journal of Allergy Clinical Immunology	BST/(3 × number of extra TPSAB1 copies)
Chovanec et al.	2022	Blood Advances	Online Calculator (https://bst-calculater.niaid.nih.gov/)

## Analysis of *KIT* D816V variant in peripheral blood

Over 90% of SM cases harbor the *KIT* D816V variant, that leads to the constitutive activation of the receptor tyrosine kinase CD117, on turn responsible for MC survival, proliferation, and differentiation (37, 38). Although *KIT* variants detection has represented a technical challenge for years due to the low fraction of pathological cells in most SM cases, significant improvements have been made in recent years. Several techniques have been developed with progressive improvement in sensitivity. Sanger sequencing was the first methodology used, affected by a low sensitivity (around 10%–20%). This was followed by the implementation of RT-PCR, which offers high sensitivity (0.05%) but lacks standardization due to the absence of a universally accepted calibrator. Peptide nucleic acid (PNA)-mediated PCR offers high specificity by blocking the amplification of wild-type sequences but has limited sensitivity in detecting low-frequency mutations (39). Therefore, it was subsequently replaced by allele-specific oligonucleotide quantitative PCR (ASO-qPCR), which allows for highly sensitive and quantitative detection of *KIT* D816V variant, making it more suitable in samples with a low mutant allele burden (40). Another standardized method for *KIT* mutation detection is droplet digital PCR (ddPCR), which partitions the target nucleic acid into thousands of nanoliter-sized droplets to enable highly accurate, absolute quantification of DNA molecules without the need for calibration standards (41). Currently, the gold standard techniques are ASO-qPCR or, preferably, droplet digital PCR (ddPCR), both of which achieve a sensitivity of 0.001% (42). Next-generation sequencing (NGS) provides a sensitivity of 1%–5%; however, it remains valuable for detecting rare, non-canonical *KIT*-activating variants (38).

Due to the lack of standardization and the known low variant burden in patients with ISM, it has been repeatedly argued that variant analysis should not be performed on peripheral blood (PB), as a negative result cannot reliably exclude the diagnosis of SM (43, 44). Nonetheless, the increased sensitivity of the methodology has shown comparable results for mutational testing on BM and PB, with a discordance rate ranging from 5% to 10% (40, 42, 45, 46). Additionally, in a recent *post hoc* analysis of the PIONEER trial on the use of avapritinib in symptomatic ISM, the central ddPCR assay method detected the *KIT* D816V variant in 37/39 (95%) of PB samples compared with 11/39 (28%) analyzed by NGS and 30/39 (80%) of PB samples analyzed locally (47), a pattern that confirms a linearity between the sensitivity of the technique and the rate of positivity. Therefore, if one wishes to initiate a patient into diagnostic procedures in this context, an accurate knowledge of the local performance of available methodologies is necessary.

A further interaction regards the correlation between BST levels and differences in detecting *KIT* variants in PB. The negative predictive value was approximately 40% in patients with BST levels between 5 and 30 ng/ml (46). In a recent retrospective study, the sensitivity of diagnostic assays for *KIT* D816V was further refined according to specific BST thresholds (≥11.5 ng/ml, ≥20 ng/ml and BST elevated due to HαT genotype) and REMA score ≥ 2. Sensitivity of PB testing was higher for BST ≥ 11.5 ng/ml and BST elevated based on genotype, while specificity was highest for BST elevated based on genotype (48). Similarly, Navarro et al. found a correlation between the percentage of *KIT* D816V-mutated cells in both PB and BM and BST levels (49).

However, tryptase represents only a minor diagnostic criterion, and a normal BST value does not exclude a diagnosis of SM, as evidenced by patients with BMM, who often present with normal BST levels (50–52). This highlights the importance of considering situations highly suspected for SM in which a direct bone marrow evaluation, including *KIT* variants analysis, should be preferred regardless of BST value (i.e., REMA score ≥ 2, MIS onset in adulthood).

## The incorporation of tryptase level in scoring models for SM

Only a small subset of SM patients (approximately 10%) present with an aggressive variant, with clinical manifestations primarily dictated by the presence of C-findings (53). In these cases, the diagnostic challenge stems from the clinical overlap with other diseases, obviously enhanced by the rarity of the disease, which can divert initial investigations towards more common causes. However, once SM is suspected, the high burden of MC infiltration, along with generally elevated tryptase levels, make the diagnosis relatively simple to establish.

Conversely, most cases of ISM present a clinical picture dominated by mediator-related symptoms, including cutaneous, gastrointestinal, musculoskeletal, and neurocognitive manifestations (54–56). When mastocytosis in the skin (MIS) appears in adult patients, it is strongly associated with a systemic form, unlike in pediatric cases, where skin lesions spontaneously regress during puberty in the vast majority of patients (57, 58). Additionally, greater than 50% of patients experience severe anaphylaxis, especially in reaction to Hymenoptera venom (59, 60). On the other hand, the prevalence on SM sets around 10% in patients experiencing anaphylaxis after Hymenoptera sting (21, 61) and 5% of all cases of anaphylaxis (62, 63). The REMA score, developed by the Spanish Network on Mastocytosis, is a diagnostic tool capable of predicting the probability of SM in patients with anaphylaxis and include BST as one of the key variables (36).

The NICAS score added the analysis of *KIT* D816V in PB, as assessed by ASO-qPCR, to the already existing parameters (slightly modifying clinical findings and BST levels) (64). In this model, the demonstration of a clonal disease in PB by the detection of *KIT* variant is clearly dominant when positive, but its incorporation is far less useful with negative results.

The ECNM proposed two algorithms for the recognition of underlying SM disorders in patient presenting with anaphylaxis. For adult patients with MIS, BM biopsy is generally recommended regardless of other findings. On the other hand, for patients without MIS, a BM biopsy is recommended when BST is greater than 25–30 ng/ml, when REMA score is ≥2, or when a *KIT* D816V variant is detected in PB (62).

Frequently, the clinical picture can be characterized by other, less specific manifestations, as gastrointestinal symptoms (i.e., epigastralgia, abdominal pain, meteorism, and diarrhea) fatigue, neurologic and/or neuropsychiatric manifestations (including depression, anxiety, headaches, and cognitive impairment such as lack of concentration). In all these cases, the recommendations for testing BST are not standardized (53–55, 65).

Several scoring systems (Table 3) have been developed to predict clonality in cases that are less characteristic than anaphylaxis. Fuchs et al. proposed a model for patients with MIS incorporating BST values as well as the presence of mediator-related symptoms (66). Among these, osteoporosis is probably the most easily identifiable and showed a prevalence of 15%–30% (67, 68). These data have justified the development of a score specific for this setting (OSTEO-score) with increased sensitivity when BST levels were corrected for the presence of HαT (69).

**Table 3:** Summary of the scoring systems evaluated in this review.

Name	Authors	Year	Description
REMA	Alvarez-Twose et al.	2012	Predicts the risk of SM in patients with anaphylaxis by evaluating the modality of reaction onset, BST levels, and gender.
NICAS	Carter et al.	2018	Adds the analysis of *KIT* D816V in PB.
	Fuchs et al.	2021	Predicts the risk of SM for patients with MIS incorporating BST values and presence of mediator-related symptom.
OSTEO	Tanasi et al.	2024	Predicts the risk of SM in patient with osteoporosis including evaluation of BST.

SM, systemic mastocytosis; BST, basal serum tryptase value; MIS, mastocytosis in the skin.

Of interest, a recent study addressed the issue of distinguishing SM patients from individuals with HαT, in view of the described overlap of some clinical symptoms. The study showed that the urinary metabolites of mediators (i.e., methylhistamine, prostaglandin F2-alpha, and leukotriene E4) were higher in SM cases than in subjects with symptomatic HαT. Although promising for their potential aid in a debated matter, these results require further validation (70).

## Conclusions

**Figure 2:**
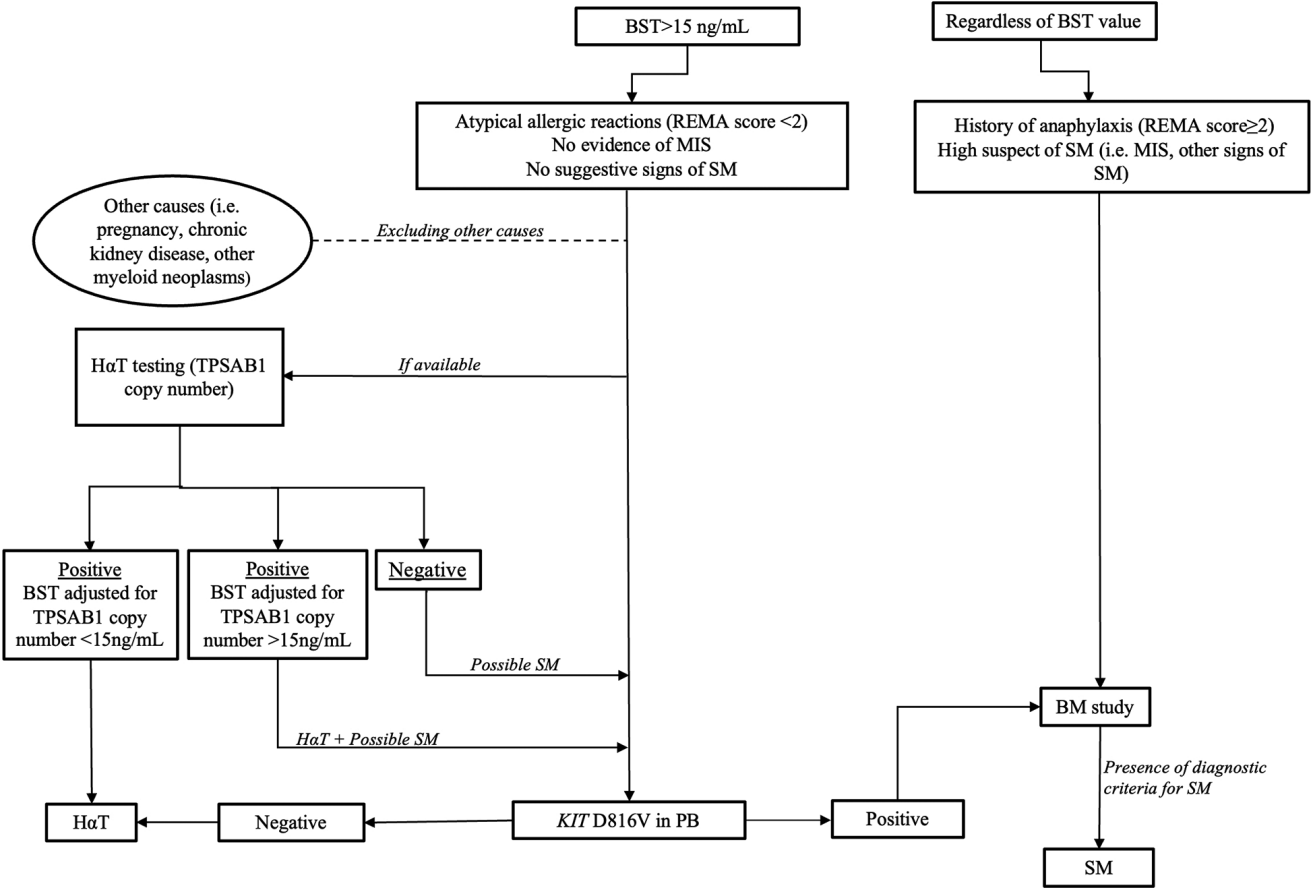
Basic algorithm for patients with suspected SM. In patients with REMA score ≥2 and/or typical skin lesions and/or suggestive signs of SM, a BM examination should be performed to confirm or exclude SM regardless of BST value. In absence of these findings within BST value >15 ng/ml, we suggested an initial evaluation of *KIT* D816V in PB and (if available) HαT testing. When KIT D816V is detectable, BM study is recommended. BST, basal tryptase level; REMA, red Espanola de Mastocitosis; MIS, mastocytosis in the skin; SM, systemic mastocytosis; HαT, hereditary alpha tryptasemia; TPSAB1, tryptase alpha/beta 1; PB, peripheral blood; BM, bone marrow.

The growing knowledge of HαT and eventually the increasingly wider use of genotyping in clinical practice could contribute to improving the diagnosis of SM, downsizing the confounding factor of high BST levels in patients with an unclear presentation of a clonal MC disorder. Moreover, the increased sensitivity of the diagnostic assays for *KIT* D816V variant is expected to improve the diagnostic algorithm in SM, especially in patients with equivocal symptoms, allowing to spare invasive procedures such as bone marrow biopsy. In this context, patients with a low REMA score (i.e., <2), no clear mediator-related symptoms (also according to the other predictive scores), and elevated BST alone could benefit from an initial evaluation of the *KIT* D816V variant in PB, along with the investigation of the HαT genotype (48). With negative *KIT* result and confirmation of HαT genotype, the suspicion of SM could be reasonably excluded (Figure 2).

Nevertheless, despite the undisputable increase in accuracy in detecting *KIT* variants in PB, major limitations remain. Several studies have shown that patients with a very low disease burden, who usually fail to meet a full diagnosis of SM and are classified as clonal mast cell activation syndrome (MMAS/cMCAS), display the greatest discrepancies in *KIT* analysis between BM and PB (49). These cases remain a grey zone that deserves further clarification because of the potentially relevant clinical implications, in terms of prevention of anaphylaxis, prescription of epinephrine and lifelong allergen-specific immunotherapy. A rationale use of BST and genetic testing in the different clinical settings is the premise to balance the application of invasive diagnostics to the actual probabilities of underlying SM disorders.
